# Performance, Behavior, and Welfare Status of Six Different Organically Reared Poultry Genotypes

**DOI:** 10.3390/ani10040550

**Published:** 2020-03-25

**Authors:** Alice Cartoni Mancinelli, Simona Mattioli, Alessandro Dal Bosco, Andrea Aliberti, Monica Guarino Amato, Cesare Castellini

**Affiliations:** 1 Department of Agricultural, Environmental and Food Science, University of Perugia, Borgo XX Giugno 74, 06124 Perugia, Italy; simona.mattioli@hotmail.it (S.M.); alessandro.dalbosco@unipg.it (A.D.B.); aliberti93.andrea@gmail.com (A.A.); cesare.castellini@unipg.it (C.C.); 2 Council for Agricultural Research and Economics—Livestock Production and Aquaculture, Via Salaria 31, 00015 Roma, Italy; monica.guarinoamato@crea.gov.it

**Keywords:** organic, chicken genotypes, behavior, productive performance, welfare status

## Abstract

**Simple Summary:**

In an extensive poultry production system, the choice of chicken strain becomes very important, because it is strictly linked to the use of outdoor space. In fact, many of the chicken strains selected for the high productive performance required by intensive production systems are not adapted to extensive rearing systems. Accordingly, in most European countries, the choice of poultry genotype usable in an organic system depends on weight gain. However, many other traits are important in the assessment of animal adaptability. The aim of this study was to investigate the behavior, the productive performance, and the welfare status of six different poultry genotypes when organically reared. The results showed that the genotypes with the best productive performance were not adapted to the organic system, because they were too static and exhibited the worst welfare status. On the contrary, strains with lower growth performance showed more active behaviors and the best welfare status. However, chickens with similar performances showed a different degree of adaptation to the extensive rearing system due to the intrinsic characteristics of the strain. In conclusion, use of an organic production system requires an equilibrium between performance and adaptability.

**Abstract:**

In alterative rearing systems, the use of outdoor space has a crucial role. It is well known that only some commercial poultry genotypes are suitable to be reared in these systems. It is necessary to find a balance between productive performance and adaptability. The aim of this study was to evaluate the productive performance, behavior, and welfare status of six poultry genotypes reared in an organic system. One hundred males/genotype (Hubbard RedJA (A), CY5XJA87 (CY), M22XJA87 (M), Ranger Classic (R1), Ranger Gold (R2), and Rowan Ranger (R3)) were reared from 1 to 81 days of age. The number of culled birds was recorded daily, whereas live weight and feed consumption were recorded weekly. Behavior evaluation was undertaken through a computerized system one week before slaughtering; the breast yield and muscle/bone ratio of the drumstick was also evaluated in refrigerated carcasses. The results showed that A and R3 had good adaptability, showing active behaviors and satisfactory productive performance 3083.6 g and 3022.1 g, respectively. Although CY and M achieved the best productive performance, they did not appear adapted to the organic system due to a higher frequency of static behaviors (rest and roost), mortality, footpad dermatitis, breast blisters, and poor feather condition.

## 1. Introduction

The EC Regulation n. 889/2008 states that, in organic production, the choice of poultry breed should take into account the capacity of the breed to adapt to local environmental conditions. However, the use of local breeds is not compulsory, and the standard does not indicate which genotypes should be employed in organic systems.

Therefore, poultry producers prefer to utilize animals with a rapid growth rate and higher breast yield. Accordingly, in Italy, big companies use both sexes of Slow-Growing genotypes and only the females of Fast-Growing genotypes (mainly Ross) in organic systems, while the males are used in intensive systems. The reason for this choice is due to the too high body weight reached by the males at 81 d. However, many authors report that even females of Fast-Growing genotypes show problems related to well-being, low meat quality, and adaptability when they are bred in organic systems due to the older slaughter age and consequently higher body weight achieved [1,2,3]. Furthermore, currently there is no univocal and official classification to identify poultry strains. Therefore, many authors [4,5] classify poultry genotypes by relying only on productive performance:

- Fast-Growing (FG) strains, adapted to intensive rearing systems, and able to reach slaughter weight in a short time (about 2.5 kg in 40 d) with very high breast yield;
- Slow-Growing (SG) strains, which represent a heterogeneous group of chickens made up of commercial strains selected by poultry companies for outdoor farming and by local poultry breeds. These are not competitive as meat-type birds.

In organic rearing systems, the use of outdoor runs has a crucial role in increasing animal welfare and the quality of products. Chickens with access to outdoor runs, compared to indoor chickens, have a higher blood antioxidant level and consequently a better oxidative response [6]. Moreover, Ponte et al. [7] assessed that the concentration of some polyunsaturated fatty acids of the n-3 series (PUFA, mainly eicosapentaenoic acid, 20:5 n-3) in the breast meat of free-range chickens is increased with respect to chickens without pasture availability. Grass intake also increases the amount of Vitamin E and n-3 PUFA in the meat of geese reared in free-range systems, compared with conventional ones [8].

The foraging behavior of chickens is strictly correlated to their kinetic activity. Mattioli et al. [9], by evaluating the effect of moderate locomotory activity in the FG and SG genotypes, showed that adaptation to exercise is very different in these two chicken strains. In particular, exercise in the FG genotype produces a worse antioxidant status with a consequent metabolic stress due to the high production of free radicals, whereas the metabolism of the SG genotype clearly showed a reliable adaptation to exercise. 

As already stated, FG genotypes [5,10] are characterized by a high live weight, which negatively influences their kinetic activity. About 60% of FG genotype, when organically reared, had several body lesions and poor feather condition [11], whereas SG chickens raised in the same conditions did not have footpad lesions or breast blisters. These studies suggest that the FG genotypes are not suitable for organic rearing systems. Indeed, these latter genotypes show an imbalance between skeletal and muscles mass that causes articular inflammations and metabolic disorders like myocardial infarcts and respiratory problems. 

Castellini et al. [11] analyzed many variables (behavior observation, oxidative status, native immunity, blood parameters, and performance) to define poultry adaptability to organic systems and showed a negative correlation between adaptation and daily weight gain. The SG birds seemed to be the most suitable strains for alternative rearing systems, whereas the FG birds showed the worst results in terms of adaptability to outdoor conditions. However, within the same subgroup of strains (e.g., SG), adaptability is not closely related to daily growth, indicating that below a certain growth level, adaptability is specifically affected by genetic strains. Adaptability to an organic system involves multiple aspects, such as physiological status, well-being and behavioral traits, requiring the analysis of numerous parameters [11].

In this composite context, it is important to find a balance between performance and adaptation. 

Based on the above-mentioned considerations, the aim of this study is to evaluate the productive performance, behavior (through a computerized video recording system), and welfare status of six poultry genotypes reared in an organic system.

## 2. Materials and Methods 

### 2.1. Birds and Farming Systems

The trial was carried out in the experimental section of the University of Perugia (Italy) from March to May 2018. The chickens were reared according to EU Regulation 834/07 and EU Regulation 889/2008 laws on animal welfare for experimental and other scientific purposes. Two breeder companies provided the studied meat type genotypes, with similar growth rate:

- Aviagen: Ranger Classic (R1), Ranger Gold (R2), Rowan Ranger (R3);
- Hubbard: Hubbard RedJA (A), CY Gen 5 × JA87 (CY), M22 × JA87 (M).

One hundred males for each meat type genotype were housed from 1 until 20 days of age in an environmentally controlled poultry house, with a temperature between 30 and 32 °C and relative humidity oscillating between 65% and 70%. At 21 days of age, the chickens were given access to outdoor space. The pasture was not treated with pesticides and contained olives trees, bushes, and hedges. 

**Figure 1:**
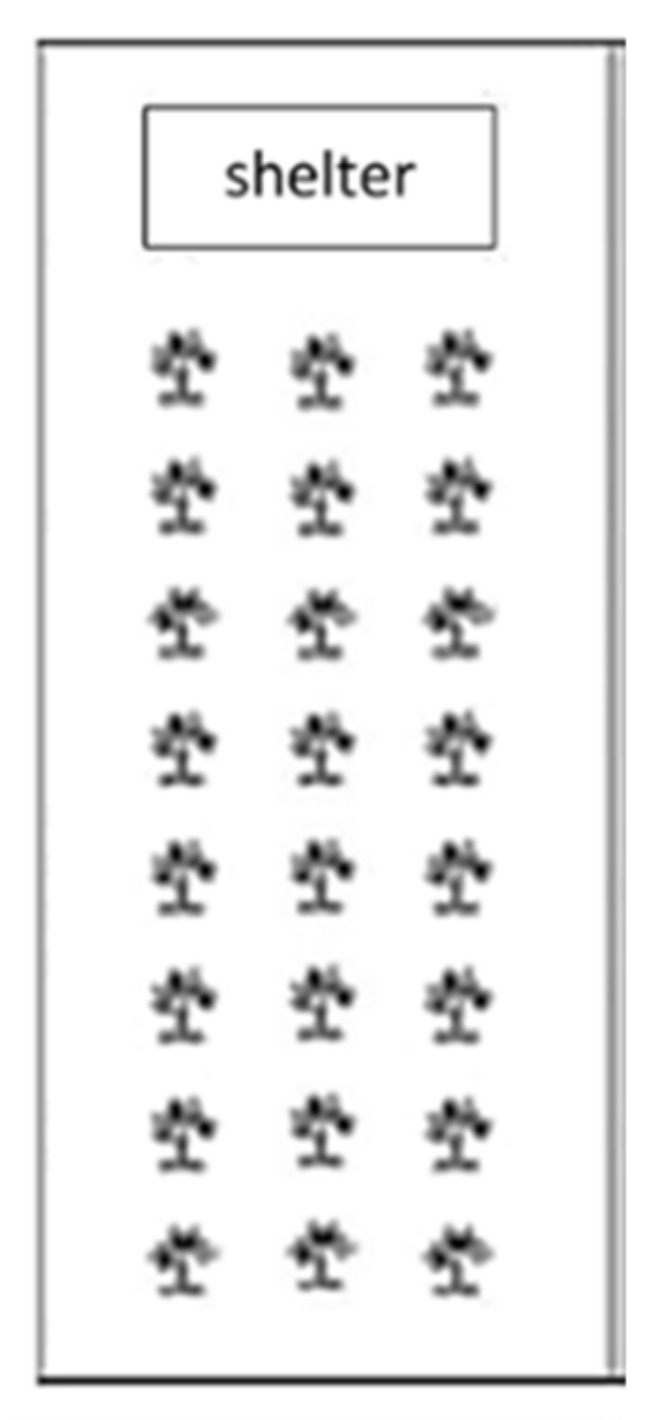
Scheme of one pen used for each genotype during the trial. In each pen, a shelter and trees are present.

Each genotype was reared separately from the others in a pen equipped with a shelter and outdoor space (Figure 1). The indoor (0.10 m^2^/bird) and outdoor (4 m^2^/bird) density of animals was chosen following organic rules (EC Regulation n. 834/2007 and 889/2008). The animals were fed ad libitum with the same organic diet (starter 1–21 d, grower 22 to 60 d, and finisher 61 d to slaughter); the diets respected the nutritional requirements of the animals as defined by NCR (1994). Water was always available and the birds were kept in shelters only during the night to protect them from predators. 

Considering that the body weight is an important factor for the animal adaptability and that males are heavier than females in this first step of study we tested only males. The animals were inspected twice a day, then the birds found dead were registered daily and the mortality rate per week was calculated (see results section), this parameter includes birds that died for natural reasons (i.e., thermal resistance, immune response, etc.). Once a week, all six genotypes were individually weighed. The feed consumption was also recorded by weighting the quantity of feed to the hand-feeders minus the residues that were weighted once a week. The feed to gain ratio (feed consumption/body weight of the group) was also calculated.

### 2.2. Carcass Traits

At 81 days of age all remaining birds were slaughtered 12  h after feed withdrawal in a commercial slaughterhouse. The animals were electrically stunned (110 V; 350  Hz) before being killed. After bleeding, the carcasses were placed in hot water (56.5 °C for 1  min) and then plucked, eviscerated (nonedible viscera: intestines, proventriculus, gall bladder, spleen, esophagus, and full crop), and stored for 24 h at 4 °C. The breasts (pectoralis major and minor) and the drumsticks of 10 animals/genotype were excised from the carcasses. The muscle/bone ratio of the drumstick and the breast yield with respect to the overall carcass were calculated.

### 2.3. Behavior Observation

The behavior evaluation was carried out using a computerized system from Noldus Technology (Wageningen, the Netherlands) consisting of two software programs: the Media Recorder 4 to record the videos and the Observer XT 14 to analyze the observations.

**Figure 2:**
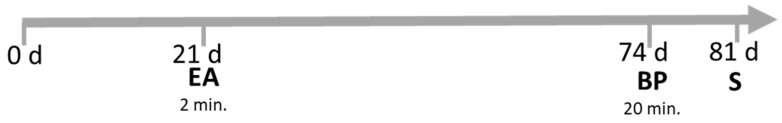
Timesheet of behavioral observations of the six poultry genotypes studied. EA Explorative Attitude, BP Behavior Pattern, S Slaughter.

The behavior observations (Figure 2) were divided into the Explorative Attitude (EA) of the animals when leaving the shelter for the first time and the Behavior Patterns (BPs) of the birds after 74 days of age.

The EA was investigated for each genotype at 21 d, the age corresponding to the first access to pasture by the animals, and this was repeated the following two days. A 5 min video was recorded for each shelter, starting from the opening of the door to allow the animals out. The videos were analyzed with the Observer XT 14 by counting the cumulative number of animals released from the shelter at the pre-established time (every 5 s). Taking into account the behavior of the different strains, only 2 min was eventually reported on.

The BPs were recorded 1 wk before slaughter (S), by recording 20 min of video covering the distance within 10 m from the shelter. Before each recording session, the operator waited 5 min for the animals to adapt to the presence of observers. The behavioral observations included Activity (active and static behaviors), Eat (feed and grass), and Comfort (stretching, grooming, etc.) (Table 1).

The videos were analyzed by three expert observers with the software Observer XT 14, through the setup of a coding scheme where the behaviors were detected and their frequencies reported.

The analysis of each video required making an observation by interrupting the video 1 time/minute for a total of 20 screen observations (*n* = 20 animals for each time). Using this procedure is possible by setting a loop option, which allows locking of the video at specific times. Every observation had a duration of 5 s, the suggested time for analysis of animal behavior expression [12,13]. The specific activity (Table 1) was expressed as a percentage of the time spent.

### 2.4. Plumage Condition, Hock Burns and Footpad Dermatitis

At 74 days of age 20 animals/genotype were subjected to evaluation of plumage. The observation focused on five parts of the body: neck, breast, back, wings, and tail. According to the Tauson method [14], a scale of values between 0 (no feathering) and 4 (intact and perfect plumage) was used.

The presence of sternal hock burn and footpad dermatitis were also taken into account through a scale of values between 0 (absence of lesions) and 1 or 2 (presence of light or deep ulcers, respectively) [15].

### 2.5. Statistical Analysis

A General Linear Model (GLM) with the effect of genetic strain was used for the analysis of the productive traits, using the STATA package [16]. Any outliers have not been removed. The statistical significance of the mortality rate was analyzed with a chi square test. 

The comparison between the different strains was performed with a multiple paired t-test (with Bonferroni correction), and the significance was established as *p* ≤ 0.05.

Categorical variables (plumage conditions, hock burns, and footpad dermatitis) were analyzed with the Kruskal–Wallis test, and the comparisons between strains were undertaken with the Dunn test.

To evaluate and compare the EA of the different strains, nonlinear models were also fitted with the following exponential equation:

EA = b_1_(1 − b_2_^time) (1)

where b_1_ represents the asymptotic value whereas b_2_ estimates the rate for reaching the asymptote. Lower value of b_2_ means higher rate for reaching the maximum value (asymptote). The goodness of fit (R^2^) was used for choosing the best model for a single strain or a group of strains.

Multivariate analysis (Principal Component Analysis, or PCA) was also performed to evaluate multiple relationships between the different traits under consideration.

## 3. Results

### 3.1. Productive Performance

Regarding the performance of the six genotypes, R1 showed the highest live weight followed by CY, M, and R2; R3 and A exhibited the lowest productive performance (Table 2). Accordingly, R1 was characterized by the highest feed intake (150.3 g/d).

The genotype that had a better feed to gain ratio was CY (2.9), followed by R1 (3.1), R2 (3.1), and M (3.4), while A and R3 showed the lowest feed to gain ratio (3.6 and 3.7, respectively).

The mortality was low in all groups; however, in particular the A, R1, R2, and R3 genotypes showed the lowest percentage, while CY and M had the highest.

The breast yield was higher in the M and R1 genotypes, whereas the muscle:bone ratio was the same (*p* ≥ 0.05) in all the genotypes studied.

### 3.2. Behavior Observations

**Figure 3:**
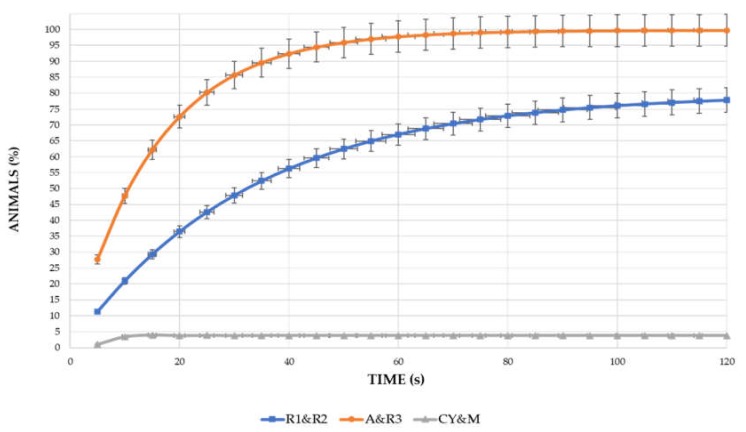
Fitted curves of Explorative Attitude in the six poultry genotypes immediately after (1–60 s) door opening (cumulative values; error bars represent 95% upper and lower CI). A: Hubbard RedJA, CY: CY5XJA87, M: M22XJA87, R1: Ranger Classic, R2: Ranger Gold, R3: Rowan Ranger.

**Figure 4:**
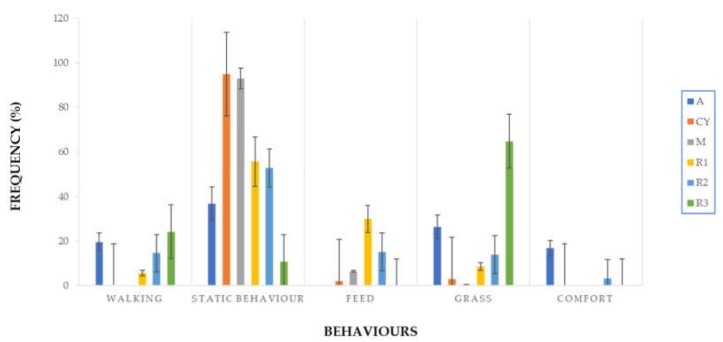
Main behavior activity of six poultry genotypes (error bars represent 95% upper and lower CI) (*n* = 20/strain). A: Hubbard RedJA, CY: CY5XJA87, M: M22XJA87, R1: Ranger Classic, R2: Ranger Gold, R3: Rowan Ranger.

The results for Explorative Attitude and other behavior traits showed that there was a high variability among the six genotypes examined (Figure 3, Table 3, and Figure 4). Three main different Exploratory Attitudes were fitted: all the models showed a very good prediction with R^2^ > 0.89 and with parameters estimated as stable and with a low asymptotic interval (b_1_) of confidence (Table 3). R3 and A, R1 and R2, and CY and M, respectively, had similar EA profiles, and their curves had the same regression parameters (b_1_ and b_2_).

R3 and A chickens had a very high Exploratory Attitude, leaving the shelter in the first seconds after door opening as indicated by the low b_2_ value. Almost all the animals of these two strains were out of the house after 30 s. R3 and A, due to their explorative capacity, were the genotypes with the highest percentage of animals out of the house (99.83%).

The R2 and R1 strains were characterized by an intermediate value of EA (78.11%), whereas only a few CY and M birds left the house (3.85%).

In Figure 4, we can see the main behavior observed as a percentage of time spent in the specific activity. Genotype A expressed the highest frequency of comfort (18%) compared with the other genotypes, while CY and M had higher levels of static behaviors (95% and 94%, respectively). R1 and R2 showed static behaviors more than 50% of the time, and they spent 30% and 16%, respectively, of their time eating feed. R3 had a higher frequency of grass intake (65%) and walking, followed by A. Overall, M and CY were the more static genotypes, showing lower rate of “active” behaviors, confirmed by the fact that, during the observations, they preferred to stay close to the shelter. 

### 3.3. Plumage Conditions, Pododermatitis, and Sternal Lesions

The six genotypes studied showed a significant difference concerning the plumage conditions among the five body regions examined, as well as in pododermatites and sternal lesions (Table 4). In general, the A strain exhibited the best body condition, showing a better score for all the parameters considered, whereas the M and CY birds showed the worst results. R1, R2, and R3 were characterized by intermediate values of plumage condition, pododermatites, and sternal lesions.

### 3.4. Principal Component Analysis

The PCA selected two components, accounting for 62.92% of the total variance (Table 5). In the first component (PC1), which explained 43.19% of the variance, static behaviors and live weight had the highest positive loadings, whereas EA and grass use the strongest negative ones (−0.51 and −0.45, respectively). The second component (PC2) explained 19.73% of the variance and was mainly influenced by breast yield, pododermatitis, and feed intake.

**Figure 5:**
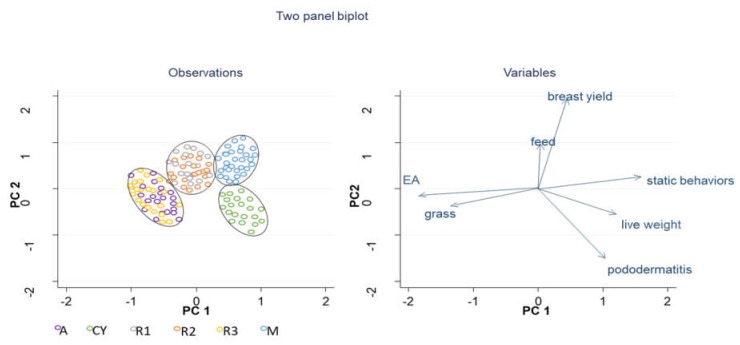
Principal Component Analysis plot showing the observations and loadings of variables, (*n* = 20/strain). A: Hubbard RedJA, CY: CY5XJA87, M: M22XJA87, R1: Ranger Classic, R2: Ranger Gold, R3: Rowan Ranger.

Thus, the different poultry genotypes were differently distributed on the factor maps (Figure 5). PCA grouped four different clusters (M & CY, A & R3, and R1 & R2). In particular, the M genotype was concentrated on the second quadrant, in which coordinates of PC1 and PC2 were positive (higher feed and breast yield); CY was scored in the third quadrant with a positive score for PC1 and negative for PC2 (static behavior, live weight, and pododermatitis). The R3 birds had negative scores for both PC1 and PC2, as well as A, and they were scored mainly on the fourth quadrants. R1 and R2 showed an intermediate position between A and R3 and M and CY.

## 4. Discussion

The results of this trial show evidence of behavioral differences among male chickens belonging to the studied genotypes (both for EA and BP). The reason for testing males is due to the fact that they usually have a higher performance than females. Considering that body weight is an important prerequisite for adaptability, if males of a specific genotype are suitable for the organic system, females are probably even more so. However, in the case of a genotype showing borderline adaptability, it may be useful to test females as well.

The A and R3 birds were the most explorative compared to the other genotypes. Indeed, they exhibited the highest EA and active behaviors, mainly in terms of frequency of walking and grass consumption. However, these strains were lighter (live weight) than the less active chickens (i.e., CY and M). The CY and M genotypes also showed higher mortality rates (0.52% and 0.61%, respectively; Table 2), probably because they are not adapted to “poor” rearing environment, which does not provide control of temperature and humidity, such as in the organic system.

Many authors have reported a negative correlation between body weight and active behaviors. Bokkers et al. [17], comparing the activity of the FG and SG genotypes under the same motivation conditions (reach feed), showed that the SG strains start to walk quickly and with a higher speed compared to the FG strains. Moreover, in the FG strains, the speed decreases with the age and with the progressive increase of body weight. 

Using a GPS monitoring device to evaluate the outdoor activity of organic chickens, it was observed that the SG birds performed more active behaviors, covering an average daily distance of 1230 m, compared to the FG birds which covered only 125 m [18].

Body weight is a good indicator of the locomotor ability of broilers, especially as age increases [19]. Accordingly, Castellini et al. [11] showed a negative correlation between daily weight gain and adaptation to an organic system, with the FG birds showing the worst results compared to the SG birds. However, the same experiment assessed that, within the same strain (e.g., SG), and below a certain level of daily gain (e.g., 40 g/d), adaptability to an extensive rearing system and daily gain are not correlated. Thus, it is important to evaluate the intrinsic characteristics and adaptability of the genotypes,

FG and SG chickens submitted to continuous and moderate exercise showed a very different physiological response [9]. After 28 d of exercise, the SG genotype showed reduced free radical generation and enhanced nonenzymatic antioxidant defenses, while the FG genotype exhibited high metabolic stress without any training along the duration of the trial and with a negative impact on the wellbeing of the birds [9]. This research is consistent with previous results and confirmed that broilers with a good level of locomotor activity and motivation to explore the outside environment (e.g., A and R3) appear more suitable for being reared in organic rearing systems, which was also confirmed by the expression of active behaviors such as walking and grass intake. 

Previous findings [20] which compared six poultry genotypes with different growth rates when organically reared confirmed that pasture intake and fatty acid metabolism are affected by poultry strain. In particular, SG strains had a high fresh forage intake and consequently higher n-3 polyunsaturated fatty acids in the breast muscle. 

The comparison of plumage conditions and body lesions in the different poultry genotypes showed that the CY and M genotypes presented the worst results for all five body regions evaluated, compared to the others. The CY group also exhibited a higher presence of pododermatitis (2.00), which was probably related to the lower kinetic activity and the later slaughter age (81 d) [21]. These latter results are in agreement with Soulliard et al. [22], who reported an increase of dirty feathers and footpad dermatitis in FG broilers reared in a mobile house compared with those in a stationary house. The results obtained in the mobile house, characterized by a poor environment (i.e., natural ventilation, etc.), suggested that FG birds should be reared in more controlled conditions, like in intensive systems.

The M genotype also showed the highest value of breast blisters (0.85), followed by the CY genotype, probably due to the high expression of “static behaviors”. The high live weight reached by these two genotypes and their less active behaviors (Figure 4) implied that they behave similarly to FG broilers. Meluzzi et al. [21] reported that the occurrence of sternal lesions and pododermatitis were dramatically higher in FG birds in comparison with SG birds and underlined the genotype effect on the behavior and welfare conditions of the birds. 

The results of our study showed the lower adaptability of the CY and M strains to the organic system. A positive correlation between active behaviors, explorative attitude, and grass intake was found; on the contrary, body weight and breast yield were strictly correlated with static behaviors and with footpad dermatitis (Figure 5).

Since more active behaviors (i.e., walk) represent an energy cost, it is hypothesized that the intensive selection for high productive performance induces a relocation of the energetic resources, with consequent modification of behavior [23]. The “resource allocation theory” confirms that genetic selection strongly modifies the allocation of dietary energy. In FG chickens most of the dietary energy is used for muscle growth, while in SG, a higher percentage of available resources is employed for other metabolic functions, like thermoregulation, movement, and feed research [24]. Accordingly, the selection for productive traits (e.g., muscle growth, breast yield) indirectly favors animals with a reduced kinetic attitude [25].

Arnould et al. [26] demonstrated that FG chickens prefer to stay near the drinkers and feeders, reducing their movements, and the environmental enrichment does not produce positive effects in welfare and walking activity because these birds use the enrichment only for sleep, with interest decreasing as age and body weight increases [27]. 

Our study also postulated that the time dedicated to feed intake could be correlated with the breast yield. Accordingly, our results show the strains with the higher breast yield (M, and partially R1 and R2) spent a lot of time close to the feeder. Many authors [28,29,30] have shown that an increase in breast yield requires high protein and amino acid levels in feed.

## 5. Conclusions

The behavior evaluation of the six genotypes evidenced that CY and M, although showing the best productive performance, did not seem well adapted to the organic system due to their higher frequency of static behaviors, higher mortality rate, more frequent footpad dermatitis, increased number of breast blisters, and poorer feather condition. Moreover, they remained close to the shelter, showing that they are not interested in using the outdoor run. Instead, R3 and A can be considered the genotypes that present the best adaptability, showing interest in exploring the pasture (e.g., EA) and high kinetic activity, alongside a satisfactory productive performance.

Hence, to define the adaptability of poultry genotypes to organic systems, besides the daily weight gain, many other aspects like behavior, welfare, and health status should be taken into account. In fact, although R2 and R3 showed a similar daily weight gain, they exhibited a different adaptability, in particular in their use of the outdoor run.

## Figures and Tables

**Table 1:** Behavior pattern analyzed within 10 meters from the shelter for each genotype.

Behavior	Description
**Activity**	Active and Static behaviors
Static behaviors	Rest: body in line with the ground, with erect head and open eyes and Roost: stay in place standing, no body movement, head erect or relaxed with open eyes
Active behaviors	Walking: moving more than three steps in one direction with upright head
**Eat**	Activities linked to eating behavior
Feed	Consume feed
Grass	Consume grass
**Comfort**	Any behavior in which the animal expresses adaptation to the environment like stretching, grooming, etc. In these behaviors the animals assume a vulnerable position (i.e., head low, lying down, in a nonalert attitude). Indeed, animals do this when they are confident with the environment and are feeling safe.

**Table 2:** Productive performance of the six poultry genotypes.

Genotype	Units	A	CY	M	R1	R2	R3	n/Genotype	Pooled SE/X^2^
Live weight at slaughter	g	3083.6 ^a^	3639.3 ^ab^	3405.0 ^ab^	3928.3 ^b^	3403.6 ^ab^	3022.1 ^a^	100	344.0
Feed intake	g/d	137.0 ^ab^	130.3 ^ab^	143.4 ^ab^	150.3 ^b^	130.3 ^ab^	141.8 ^a^	100	20.0
Feed:gain ratio		3.6 ^b^	2.9 ^a^	3.4 ^ab^	3.1 ^a^	3.1 ^a^	3.7 ^b^	100	1.0
Mortality	% (n)	0.17 ^a^ (2)	0.52 ^b^ (6)	0.61 ^b^ (7)	0.09 ^a^ (1)	0.17 ^a^ (2)	0.17 ^a^ (2)	12	2.06 *
Breast yield	%	18.8 ^a^	20.1 ^a^	28.1 ^b^	26.0 ^b^	22.7 ^a^	21.8 ^a^	10	2.5
Muscle/bone	%	2.5	2.7	2.5	3.0	2.6	2.7	10	0.2

On the same row a–c = *p* ≤ 0.05. *—X^2^ value. A: Hubbard RedJA, CY: CY5XJA87, R1: Ranger Classic, R2: Ranger Gold, R3: Rowan Ranger, M: M22XJA87.

**Table 3:** Main different Explorative Attitudes among the genotypes studied (*n* = 20/strain).

Strain	b_1_	b_2_	Upper 95%b_1_	Lower 95%b_1_	R^2^	Root MSE
CY & M	3.85 + 0.31 ^a^	−0.88 + 0.03	2.5	4.3	89.9	1.89
R1 & R2	78.11 + 9.22 ^b^	0.97 + 0.05	65.0	93.0	96.2	9.42
A & R3	99.83 + 2.95 ^c^	0.94 + 0.05	94.0	103.0	98.8	9.44

On the same column a–c = *p* ≤ 0.05. b_1_: represents the asymptotic value; b_2_ estimates the rate for reaching the asymptote. A: Hubbard RedJA, CY: CY5XJA87, M: M22XJA87, R1: Ranger Classic, R2: Ranger Gold, R3: Rowan Ranger.

**Table 4:** Comparison of plumage conditions and body lesions in the six poultry genotypes (*n* = 20/strain).

Genotypes	A	CY	M	R1	R2	R3	X^2^
Neck	4.00 ^b^	2.75 ^a^	3.00 ^a^	3.71 ^b^	3.57 ^b^	3.71 ^b^	16.43
Breast	2.14 ^b^	1.71 ^ab^	1.25 ^a^	1.57 ^ab^	1.71 ^ab^	1.71 ^ab^	3.67
Wings	3.43 ^c^	2.25 ^ab^	2.00 ^a^	3.29 ^bc^	3.57 ^c^	3.29 ^bc^	16.56
Back	4.00 ^b^	3.00 ^a^	3.25 ^a^	4.00 ^b^	4.00 ^b^	3.71 ^b^	27.17
Tail	3.43 ^b^	1.75 ^a^	2.50 ^a^	3.43 ^b^	3.43 ^b^	3.14 ^b^	13.85
Pododermatitis	0.00 ^a^	2.00 ^b^	1.25 ^a^	1.43 ^a^	0.43 ^a^	0.14 ^a^	13.83
Sternal lesions	0.00 ^a^	0.70 ^b^	0.85 ^b^	0.34 ^ab^	0.14 ^ab^	0.2 ^ab^	3.07

a–c on the same line means *p* < 0.05. A: Hubbard RedJA, CY: CY5XJA87, M: M22XJA87, R1: Ranger Classic, R2: Ranger Gold, R3: Rowan Ranger.

**Table 5:** Principal component loadings, Eigen value, and variance.

Trait	PC1	PC2
Static behavior	**0.51**	0.07
Live weight	0.39	−0.19
Pododermatitis	0.31	**−0.52**
Breast yield	0.14	**0.68**
Feed	0.01	0.47
Grass	−0.45	−0.17
EA	**−0.52**	−0.08
Eigen value	3.03	1.36
% Variance explained	43.19	19.73
Cumulative variance explained	62.92	

Loadings ≥0.50 or ≤−0.50 are bolded.
